# Impact of Brewers’ Spent Grain-Containing Biscuit on Postprandial Glycaemic Response in Individuals with Metabolic Syndrome: A Crossover Randomised Controlled Trial

**DOI:** 10.3390/nu16060909

**Published:** 2024-03-21

**Authors:** Yujing Xu, Zi Ning Leong, Weijia Zhang, Xinrui Jin, Jia Wen Kong, Gregory Chung Tsing Chan, Jung Eun Kim

**Affiliations:** 1 Department of Food Science and Technology, Faculty of Science, National University of Singapore, 2 Science Drive 2, Singapore 117543, Singapore; yujingxu@u.nus.edu (Y.X.); leong.zining@nus.edu.sg (Z.N.L.); zhangweijia0817@163.com (W.Z.); e0950404@u.nus.edu (X.J.); kongjw@u.nus.edu (J.W.K.); 2 Saw Swee Hock School of Public Health, National University of Singapore, Singapore 117549, Singapore; ephgchan@nus.edu.sg

**Keywords:** dietary fibre, dietary protein, brewery by-product, *Rhizopus oligosporus*, subjective appetite, postprandial lipid panel response

## Abstract

Brewers’ spent grain (BSG) is a fibre and protein-rich by-product of beer-brewing. Fermenting BSG with *Rhizopus oligosporus* can further increase its content of soluble fibre, protein and certain antioxidants. Since nutrients rich in BSG can improve postprandial glycaemic response, this study assessed the postprandial glucose response (PPGR) and postprandial insulin response (PPIR) controlling effect of consuming 30% wheat flour substituted biscuits with autoclaved BSG (ABSG) or fermented BSG (FBSG) in individuals with metabolic syndrome (MetS). The effect on postprandial lipid panel, breath hydrogen (H_2_) and methane (CH_4_) concentration and subjective appetite response was also examined. Fifteen subjects with MetS participated in this crossover randomised controlled trial, and blood was collected at 9 time-points for 4 h after consumption of control biscuits (Control), ABSG and FBSG. A significant interaction effect was observed (*P*_interaction_ = 0.013) for the glucose time-points concentration. At 180 min, the glucose concentration was lowered after the consumption of ABSG (*p* = 0.010) and FBSG (*p* = 0.012) compared to the Control. Moreover, the FBSG resulted in a significantly lower glucose incremental area under curve (iAUC) compared to the Control (*p* = 0.028). Insulin level was also lowered at 180 min after the ABSG (*p* = 0.010) and FBSG (*p* = 0.051) consumption compared to the Control. However, no difference was noted for postprandial lipid panel, breath H_2_ and CH_4_ concentration and subjective appetite response. In conclusion, the consumption of BSG-incorporated biscuits can attenuate PPGR, and fermented BSG incorporation conferred a further PPGR controlling benefit.

## 1. Introduction

Postprandial hyperglycaemia is an important contributor to type 2 diabetes mellitus (T2DM)-related complications, such as cardiovascular disease (CVD) [1]. Moreover, it has been reported that postprandial glucose response (PPGR) could serve as a more effective predictor of cardiovascular events compared to fasting glucose concentration [2]. Another factor closely associated with the development and progression of T2DM and CVD is metabolic syndrome (MetS) [3,4], a pathophysiological clustering of chronic disease risk factors including abdominal obesity, hypertension, hyperglycaemia and dyslipidaemia [5]. Individuals with MetS exhibit a higher risk of impaired PPGR, primarily due to insulin resistance, a key characteristic of MetS [6,7]. Consequently, establishing interventions that regulate PPGR in this population is crucial.

Biscuits are widely consumed due to their ease of consumption, long shelf life, high affordability, and palatability [8,9]. However, they are commonly formulated with refined grains and tend to cause a rise in PPGR [10]. Among the various factors affecting PPGR, fibre has been proven by numerous studies to modulate PPGR by slowing gastric emptying, promoting insulin release, and decreasing glucagon release [11,12,13,14]. Brewer’s spent grains (BSG), a fibre-rich by-product of the beer-brewing process, contains 41–59% dietary fibre (dry weight basis) [15,16], thus, incorporating BSG into biscuits may improve the nutritional profiles in biscuits and promote a desirable PPGR after consumption [17,18]. Moreover, previous studies have also indicated that the extract of dietary protein and phenolic compounds from BSG exhibits potential in regulating PPGR [19,20,21]. However, since the primary dietary fibre in BSG is insoluble fibre, the dietary protein and phenolic compounds are physically trapped in the insoluble dietary fibre matrix [22]. This may reduce the bioaccessibility of these nutrients, thereby limiting the effectiveness of BSG in regulating PPGR after consumption.

Our recent study showed that the solid-state fermentation of BSG with *Rhizopus oligosporus* (RO) could break down insoluble dietary fibre into soluble dietary fibre [18]. Soluble dietary fibre can be further fermented by gut microbiota to promote the secretion of short-chain fatty acids [23], which in turn will regulate glucose metabolism by stimulating the secretion of glucagon-like peptide-1 and peptide tyrosine tyrosine, and upregulating the expression of glucose transporter-4 [24,25,26]. The consumption of fermented BSG-incorporated biscuits may therefore provide a further improvement on PPGR.

At present, limited clinical research has been conducted to verify the potential benefits of BSG and fermented BSG-containing biscuits consumption on PPGR regulation, particularly in individuals with MetS. Hence, the aim of the current study was to recruit individuals with MetS into a crossover randomised controlled trial (RCT) to explore the PPGR and postprandial insulin response (PPIR) after consuming BSG and fermented BSG-containing biscuits. The effect on the postprandial lipid panel, breath H_2_ and CH_4_ concentration and subjective appetite response were also examined as secondary outcomes in this research.

## 2. Materials and Methods

### 2.1. BSG Fermentation and Biscuits Baking

The fermentation of BSG and formulation of biscuits were carried out as per our previous work [18] and briefly elaborated below. BSG was provided by Brewerkz Brewing Co. (Singapore) and sterilised at 121 °C for 15 min to produce autoclaved BSG. Ragi tempe starter (PT. Aneka Fermentasi Industri, Bandung, Indonesia), a mixture of RO spores and rice flour, was added to autoclaved BSG cooled to room temperature at 4% of wet autoclaved BSG basis and subsequently incubated at 37 °C for 72 h to obtain fermented BSG. All autoclaved BSG and fermented BSG were freeze-dried (Lyovapor L-300, BUCHI, Singapore) to a water content of less than 5% and stored at −20 °C until use.

The control biscuits (Control), autoclaved BSG-containing biscuits (ABSG) and fermented BSG-containing biscuits (FBSG) were made with the same base recipe, with ABSG and FBSG made with a 30% wheat flour substitution of autoclaved or fermented BSG powder, respectively. The base recipe contained 180 g wheat flour, 10 g corn flour, 50 g fine sugar, 1 g salt, 1 g baking soda, 55 g sunflower oil and 70 g whole egg. Additionally, 15 g water was added to the ABSG and FBSG recipe to improve dough binding. The whole egg, sunflower oil and fine sugar were mixed at speed 2 for 1 min using a stand mixer flat beater (KitchenAid Classic, Battle Creek, MI, USA) before the remaining ingredients were mixed at speed 1 for 90 s. The dough was sheeted to a 3 mm thickness, sliced into 55 mm squares, and baked at 160 °C in a convection oven (Anna, Unox, Cadoneghe, Italy) for 20 min. Upon cooling to room temperature, all the biscuits were stored at 4 °C until use.

The nutritional profile of the test biscuits was measured and detail methods are described in previous work [18]. The results were converted to wet basis and tabulated in Table 1.

### 2.2. Study Design and Subject Recruitment

The study was registered at clinicaltrials.gov (NCT05421780) and approved by the National University of Singapore Institutional Review Board (NUS-IRB-2022-089) on 25 May 2022.

The recruitment of subjects with MetS aged 35–85 years old took place from June to December 2022 in Singapore by placing posters, sending emails, and advertising through word-of-mouth. The criteria for MetS was in accordance to the National Cholesterol Education Program Adult Treatment Panel III (NCEP-ATP III), where subjects that meet any three of the following five criteria were defined as with MetS: (1) waist circumference > 102 cm (male), >88 cm (female), and for the Asian population, >90 cm (male), >80 cm (female); (2) fasting glucose concentration ≥ 5.6 mmol/L or on known medication for blood glucose control; (3) triglyceride (TG) ≥ 1.7 mmol/L or on known medication for TG control; (4) high-density lipoprotein cholesterol (HDL-C) < 1.0 mmol/L (male), <1.3 mmol/L (female); (5) systolic or diastolic blood pressure > 130/85 mmHg or on known medication for blood pressure control. The exclusion criteria to reject subjects whose current lifestyle may impact the outcomes of interest was as follows: (1) significant change in weight (≥3 kg) over the past 3 months; (2) allergy to barley, wheat, corn, egg, or any other ingredients found inside the biscuits; (3) acute illness at study baseline; (4) exercising vigorously over the past 3 months; (5) following any restricted diet (e.g., vegetarian); (6) smoking; (7) have a daily intake of more than 2 alcoholic drinks per day; (8) prescribed and taking antihypertensive/cholesterol-lowering/T2DM medication for less than 3 years prior to the intervention participation; (9) taking fermented food regularly or any probiotic/prebiotic supplements; (10) consumption of antibiotics over past 3 months; (11) pregnant, lactating, or planning pregnancy in the next 6 months; (12) current daily intake of brown rice or wholemeal products comprising more than 6 servings; (13) current daily intake of vegetables comprising more than 2 servings.

**Figure 1:**
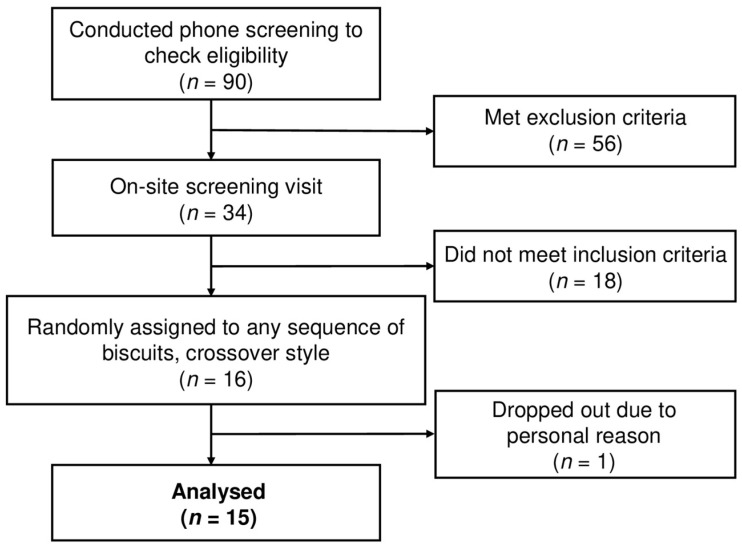
Flow chart of the clinical trial enrolment, randomisation, allocation, and analysis.

Sixteen subjects were recruited and provided their written informed consent. Following the consent, subjects were randomly assigned to the intervention order using STATA (Version 13, StataCorp LC, College Station, TX, USA) by an unblinded staff. Fifteen subjects finished the entire intervention and one subject dropped out due to personal reasons. The CONSORT flow diagram for our study is as shown in Figure 1.

### 2.3. Study Design

**Figure 2:**
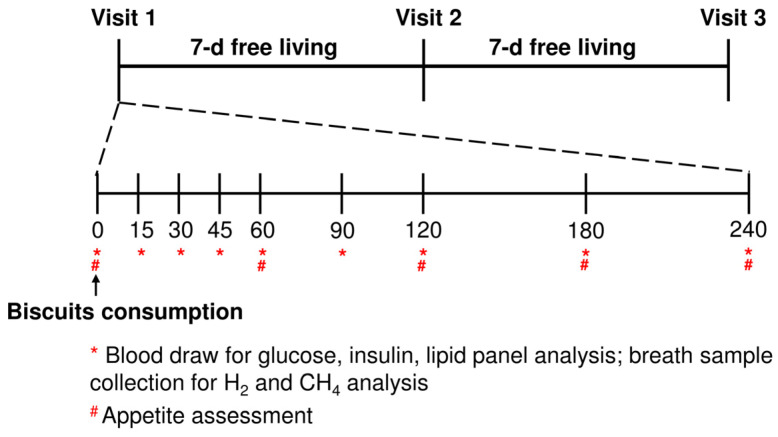
Study design timeline of a crossover design, 3-arm randomised controlled trial. Fifteen subjects were randomly assigned to the intervention order of consuming Control, FBSG and ABSG. Control: control biscuit; ABSG: autoclaved brewers’ spent grain-containing biscuit; FBSG: fermented brewers’ spent grain-containing biscuit.

The study procedure is depicted in Figure 2. This was a randomised, crossover, double-blinded study with three visits and a 7 day washout period was set between each visit. Prior to the test visit, subjects were instructed to fast for more than 10 h and to avoid taking high-fibre meals and alcohol the day before. Moreover, anti-diabetic, cholesterol-lowering and anti-hypertensive medications were also stopped. After the subjects arrived at the National University Health System Investigational Medicine Unit, anthropometric measurements and blood pressure were taken by trained staff. Subjects lay in a phlebotomy bed for the entirety of the visit, and blood collection was completed through an indwelling venous cannula inserted into the antecubital vein on the forearm, with a saline flush conducted every time a blood aliquot was taken. After fasting, blood and breath were taken (time = 0 min) and subjects were asked to consume a pack of biscuits (90 g) within 15 min. Additionally, a cup of plain water (250 mL) was provided to facilitate the consumption of the biscuits, although complete consumption of water was not obligatory. As the main aim of this study was to mimic a typical eating pattern incorporating whole BSG, the biscuits provided were standardised based on weight (90 g) instead of matching available carbohydrates (ACHO). The timer was started on their first bite, and blood samples were taken at time = 15, 30, 45, 60, 90, 120, 180 and 240 min. A heating pad was used to warm the subjects’ forearm throughout the entire visit duration to avoid coagulation in the cannula.

### 2.4. Anthropometric and Blood Pressure Measurement

The height and weight were measured using an electronic scale stadiometer (BSM370, Biospace Co., Ltd., Seoul, Korea), with body mass index (BMI) calculated by weight/height2. The waist circumference (WC) was taken as the midpoint between the lower margin of the last rib and top of the iliac crest according to the World Health Organisation STEPwise Approach to Surveillance protocol [27]. Subjects lay on the phlebotomy bed for 5 min before taking resting blood pressure (Omron HEM-7121, Kyoto, Japan). All the measurements were taken in duplicates.

### 2.5. Blood Sample Processing and Biochemical Analysis

All blood samples were drawn by certified phlebotomists. At each timepoint, 2 mL and 3 mL of blood was collected in a fluoride tube and serum separator tube respectively (Becton Dickinson, Franklin Lakes, NJ, USA). Fluoride tubes and serum separator tubes were kept at 4 °C and outsourced to Quest Laboratories Pte Ltd. (Singapore) for glucose, insulin and lipid panel analysis. The glucose and lipid panel was analysed using ADVIA 1800 and ADVIA Chemistry XPT (Siemens, Munich, Germany). Glucose concentration was determined using the enzymatic method with hexokinase and glucose-6-phosphate [28]. Insulin concentration was measured through electro-chemiluminescence immunoassay using a Cobas 6000 (e601) analyser (Roche, Basel, Switzerland). Total cholesterol (TC) and HDL-C concentrations were quantified using an enzymatic method based on the Trinder reaction and the Fossati three-step enzymatic reaction, while a Trinder endpoint was used to determine TG concentration [29]. After determining the TC, HDL-C, and TG, low-density lipoprotein cholesterol (LDL-C) was calculated using the Friedwald formula [30].

### 2.6. Breath Analysis

The breath samples were collected by trained staff. Subjects were required to continuously blow into a vented polyethylene bag with a medium-resistance leak at time = 0, 15, 30, 45, 60, 90, 120, 180 and 240 min. Subsequently, a 25 mL breath sample was drawn steadily into a syringe. Twenty-mL was injected into a Quintron breath analyser (BioMedix Singapore Pte Ltd., Singapore) to analyse the concentration of hydrogen (H_2_) and methane (CH_4_) present to assess gastrointestinal bacteria fermentation.

### 2.7. Appetite Sensation

Subjective appetite was evaluated via the Visual Analogue Scale (VAS) of appetite proposed by Flint [31]. Responses were collected every 60 min (time = 0, 60, 120, 180 and 240 min). The VAS contained questions regarding subjects’ feelings of hunger, desire to eat, prospective consumption and fullness. For each query, answers were collected through a scale represented as a 100 mm long horizontal line, with no gradation. The subjects were asked to mark a cross at the point of the scale to assess their sensation at each timepoint. After collection, a ruler measurement from the left end of the line (0 mm) to the right end (100 mm) was performed to obtain the appetite score.

### 2.8. Power Calculation and Statistical Analysis

The primary outcome of interest in this research is the difference in postprandial glucose concentration after consuming BSG-containing biscuits, compared to the Control. Recent research reported that the plasma glucose concentration area under the curve_0–120min_ (AUC_0–120min_) was lower with a BSG-containing high fat meal (2613 ± 193 a.u., mean ± SD), compared to the same meal without BSG (2936 ± 139 a.u., mean ± SD) in the animal model [32]. In addition, an in vitro digestion study reported that total glucose release AUC_0–360 min_ was lower with RO fermented okara-containing biscuits (166.8 ± 7.7 mg/min, mean ± SD) compared to the same biscuits without okara (187.9 ± 17.5 mg/min, mean ± SD) [33]. For the current study, presuming the proposed experiment yields similar results as previous studies, ≥6 subjects will provide ≥90% power at α = 0.05 (two-tailed) to statistically confirm a similar difference. Due to the different study types (animal model vs. in vitro vs. human) and study design, a minimum recruitment of 15 subjects is required, with a maximum recruitment of 19 subjects to account for a 20% dropout.

Two researchers were involved in data collation to ensure accuracy. A blinded researcher proceeded with the data analysis and was only unblinded after the completion of statistical analysis. The change from the baseline of serum glucose, insulin and lipid lipoprotein concentration was plotted against time. Subsequently, the positive incremental area under the curve (iAUC) of glucose, insulin and TG were calculated with GraphPad Prism 9.3.0 software (GraphPad Software Inc., La Jolla, CA, USA) by using the trapezoidal rule formula. As TC, HDL-C and LDL-C may decrease before rising over the entire postprandial duration, negative iAUC for the postprandial lipid lipoprotein response was also calculated. The subjective appetite assessment score was also plotted against time. All data were checked for normality distribution using a Shapiro–Wilk test and were logarithmised if necessary. Two-way repeated measures ANOVA were used to examine the effect of time, intervention and their interaction (time × intervention). The Wilcoxon test considering Bonferroni correction for multiple comparisons was used to determine the simple main effect when the interaction effects were significant. One-way repeated-measures ANOVA and pairwise comparison with the Bonferroni test was conducted to assess the effect of intervention among groups in iAUC. The figures were drawn using GraphPad Prism 9.3.0 software (GraphPad Software Inc., La Jolla, CA, USA). IBM SPSS Statistics 20 (SPSS Inc., Chicago, IL, USA) was used for all statistical analysis, and a *p* value < 0.05 was considered statistically significant. The data of the clinical trial are presented as mean ± SEM.

## 3. Results

### 3.1. Subjects’ Baseline Characteristics

Subjects’ baseline characteristics are shown in Table 2. Fifteen middle-aged and older adults (mean age: 63 ± 10 years old, 10 males and 5 females) participated in this study and all of them were diagnosed as MetS. Among all subjects, 8, 6 and 1 subjects met 3, 4, 5 MetS components respectively.

### 3.2. Postprandial Glucose and Insulin Response

**Figure 3:**
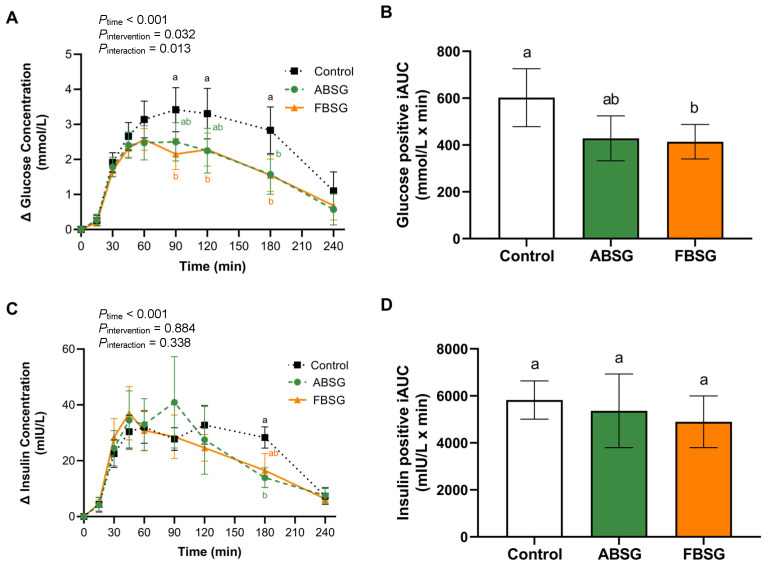
(**A**) Change from baseline of postprandial glucose concentration. (**B**) Positive iAUC of glucose. (**C**) Change from baseline of postprandial insulin concentration. (**D**) Positive iAUC of insulin. *p* values were determined using two-way repeated measures ANOVA (**A**,**C**) or one-way repeated measures ANOVA (**B**,**D**). Groups with different letters indicate a statistical difference. Control: control biscuit; ABSG: autoclaved brewers’ spent grain-containing biscuit; FBSG: fermented brewers’ spent grain-containing biscuit.

The changes in postprandial glucose and insulin concentration from 0 min and positive iAUC are shown in Figure 3. For PPGR (Figure 3A), a significant interaction effect was observed (*P*_interaction_ = 0.013) for glucose time-points concentration. Pairwise comparison showed that at time-points 90 and 120 min, FBSG resulted in a significantly lower glucose concentration compared to the Control (FBSG vs. Control: 90 min: 2.15 ± 0.44 mmol/L vs. 3.42 ± 0.62 mmol/L, *p* = 0.010; 120 min: 2.28 ± 0.47 mmol/L vs. 3.31 ± 0.72 mmol/L, *p* = 0.041). Moreover, at 180 min, both ABSG and FBSG showed a significantly lower glucose concentration compared to the Control (ABSG vs. Control: 1.57 ± 0.57 mmol/L vs. 2.83 ± 0.67 mmol/L, *p* = 0.010; FBSG vs. Control: 1.54 ± 0.46 mmol/L vs. 2.83 ± 0.67 mmol/L, *p* = 0.002), with no significant difference between ABSG and FBSG. As compared to the Control consumption, FBSG consumption resulted in a significantly lower positive iAUC for glucose (FBSG vs. Control: 413.9 ± 73.8 mmol/L × min vs. 602.0 ± 123.4 mmol/L × min, *p* = 0.028) while no difference between ABSG and the Control was observed (*p* > 0.05) (Figure 3B).

Regarding the PPIR (Figure 3C), no interaction effect was observed (*P*_interaction_ = 0.34). At 180 min, the concentration of insulin was significantly higher in the Control than ABSG (Control vs. ABSG: 28.32 ± 3.81 mIU/L vs. 13.91 ± 3.56 mIU/L, *p* < 0.001), and FBSG tended to have a lower insulin concentration compared to the Control (FBSG vs. Control: 16.55 ± 6.08 mIU/L vs. 28.32 ± 3.81 mIU/L, *p* = 0.051). However, no difference was noted in positive iAUC for insulin (*p* > 0.05) (Figure 3D).

### 3.3. Postprandial Lipid Panel Response

The responses in the postprandial concentration of TC, HDL-C, LDL-C, and TG concentrations from baseline are shown in Supplemental Figure S1 and the negative iAUC of TC, HDL-C and LDL-C, as well as positive iAUC of TG, are summarised in Supplemental Table S1. No difference was observed for any of the outcomes.

### 3.4. Breath Analysis

The changes in postprandial breath H_2_ and CH_4_ are shown in Supplemental Figure S2 and no difference was observed for postprandial breath H_2_ and CH_4_ after consuming the 3 types of biscuits (*p* > 0.05).

### 3.5. Subjective Appetite Assessment

The effect on the postprandial subjective sensation of hunger, desire to eat, prospective consumption and fullness are shown in Supplemental Figure S3 and there was no difference on those measurements between the Control, ABSG and FBSG (*p* > 0.05) at all time-points.

## 4. Discussion

While BSG and fermented BSG could serve as fibre-rich ingredients to be incorporated into food products to enhance their nutritional value, research into its effect on PPGR is lacking. In addition, there are limited studies exploring the potential of diet modifications in improving PPGR in the MetS population, who experience an impaired glucose metabolism. Our findings suggest that the consumption of biscuits with a 30% substitution of wheat flour with BSG improves PPGR in individuals with MetS and a further benefit on PPGR was offered by FBSG-containing biscuits consumption.

A significant difference was found in PPGR between the Control and BSG-containing biscuits consumption. Both ABSG and FBSG showed a more than 16% lower in glucose iAUC (29% and 31%, respectively), which is considered clinically relevant [34], although the decrease in ABSG is statistically insignificant. The decrease in the PPGR in the FBSG and ABSG could be attributed to the lesser ACHO content in the BSG-containing biscuits (20% in ABSG and 23% in FBSG). However, a previous study that substituted whole maize flour into biscuits which resulted in an ACHO reduction (18%), similar to our study here, only showed a 14% decrease in glucose AUC [35]. This implies that the presence of other compounds within BSG may also contribute to the decrease in PPGR observed. In a prior study by Ullah, et al. [36], the consumption of BSG extract-based breadsticks with matched ACHO showed an improvement in PPGR at 90 and 120 min compared to the consumption of plain breadsticks in individuals with impaired glucose tolerance. As a high-fibre food by-product, BSG-containing biscuits showed a significantly higher total dietary fibre content [18] and this dietary fibre is able to increase the viscosity of gastric contents and slow down gastric emptying, leading to a delayed and reduced glucose absorption [37]. Additionally, the antioxidants present in BSG, such as phenolic compounds and bioactive peptides, also contribute to the improvement in PPGR regulation. These antioxidants function as α-glucosidase inhibitor, which prevents the breakdown of complex carbohydrates into glucose [18]. On the other hand, these antioxidants could promote glucose consumption and glycogen synthesis, enhancing the utilisation of glucose [38].

Moreover, FBSG demonstrated a further improved PPGR regulating effect, exhibited through a significantly lower positive iAUC compared to Control, whereas ABSG did not. This outcome is attributed to a portion of the insoluble dietary fibre being broken down to soluble dietary fibre during the fermentation process by certain extracellular carbohydrases secreted by RO, such as cellulase, endocellulase, and hemicellulose [33]. Soluble dietary fibre demonstrates a greater ability to inhibit glucose absorption in the intestine compared to insoluble dietary fibre because of its high viscosity and gel-forming properties [39]. A previous RCT evidenced that incorporating β-glucan, a soluble fibre also present in BSG, into tortillas improved PPGR [40].

Nevertheless, a recent study found that there was no difference in PPGR among healthy subjects who consumed either 100 g of 40% autoclaved or RO fermented okara-substituted biscuits, which is also a dietary fibre-rich food by-product, compared to those who consumed 100 g of control biscuits [41]. The difference in findings may be caused by the different health conditions of the subjects involved in each study. The dietary fibre rich food by-products may be more effective in managing PPGR in individuals with MetS because they have impaired glucose tolerance and insulin resistance, which can lead to higher PPGR [42]. This finding is supported by the result of a systematic review and meta-analysis, which concluded that metabolically impaired individuals showed a more significant improvement in PPGR after consuming a low glycaemic index breakfast compared to healthy individuals [6].

In our results, ABSG and FBSG showed a lower insulin concentration at 180 min in comparison to Control, and the iAUC was lower although statistically insignificant. Dietary fibre is extensively credited as the main reason for the improvement of PPIR, as its consumption is associated with increased insulin sensitivity [43]. Additionally, phenolic compounds present in BSG could also play a supporting role. Reactive oxygen species generated from glucose metabolism could trigger the secretion of glucose-stimulated insulin [44] and phenolic compounds may combat these reactive oxygen species. Despite this, an overall insignificant change in insulin iAUC is likely due to the high dietary protein content in ABSG and FBSG compared to the Control. Dietary protein is recognised as insulinotropic since it stimulates insulin secretion by producing insulin-related amino acids during digestion, such as branched-chain amino acids [45]; therefore, this effect may offset the benefit provided by dietary fibre and phenolic compounds.

We also examined the postprandial lipid-panel response after biscuits consumption and no effect on postprandial TG response is observed, even though consumption of soluble fibre has been shown to enhance postprandial TG response [46]. That being said, another study conducted by Bourdon, et al. [47], also showed no difference in postprandial TG response after consumption of pasta made with a 40% barley flour substitution compared to pasta made with wheat flour. The reason for such conflicting results could be due to the amount of soluble dietary fibre present in the biscuits. Previous studies have shown that a minimum of 10 g of soluble dietary fibre is required to reduce postprandial TG response [48], while the soluble dietary fibre content in our biscuits is noted to be below 1.2 g [18]. In addition, the 4-h duration of the postprandial study could also explain the discrepancy in results, as lipid metabolism requires a longer postprandial duration of around 6-h [46].

We anticipated that the BSG-containing biscuits would increase the concentration of breath H_2_ and CH_4_, as BSG-containing biscuits contain more non-digestible carbohydrates, such as cellulose and hemicellulose, which can be fermented by gut microbiota [49]. However, in contrast, no difference was observed and it is possibly explained by the relatively short postprandial duration in this study. As the primary dietary fibre in the BSG is insoluble, it cannot be rapidly fermented by gut microbiota [50]. According to a previous study by Belobrajdic et al. [51], it takes at least 270 min for 121 g of wholegrain bread to reach the large intestine. In line with this study, results from O’Connor and Campbell [52] found that after consuming a 15 g insoluble dietary fibre-enriched snack bar, H_2_ levels increased after 240 min, and a significant difference with a plain snack bar was only observed after 360 min.

A previous study demonstrated that arabinoxylans, the main oligosaccharide in BSG (21–28% on dry weight basis), exhibit a great water holding capacity, thereby increasing digested food viscosity and prolonging stomach distension [53]. With these characteristics, it was expected that the consumption of BSG-containing biscuits will suppress appetite; however, no difference was observed in this study. This may be attributed to the fact that the subjects enrolled into this study are relatively older adults and may exhibit a slower gastric emptying due to increased pyloric motility, greater gastric antral area, and decreased perception of gastric distension [54]. Moreover, older adults experience a decrease in appetite due to the elevated secretion of appetite-suppressing peptides in conjunction with a decreased secretion of appetite-stimulating peptides [55]. Hence, any changes in postprandial appetite for the older adults may be less obvious when reported through a subjective appetite assessment.

The novelty of the study lies in the incorporation of BSG or fermented BSG into food products and the subsequent assessment of its PPGR and PPIR controlling effects. These findings provide clinical evidence to facilitate the development of functional food with BSG that can assist in improving glucose metabolism while simultaneously addressing the environmental challenge of BSG disposal. Another key strength of this study lies in the recruitment of individuals with MetS, as these individuals specifically require dietary strategies for PPGR regulation. However, present limitations in our study should also be noted. Although 15 subjects provided more than 90% statistical power, the sample size remains relatively small. Therefore, future studies should consider enlarging the study population to enhance the generalizability of the findings across broader demographic groups. Additionally, as the main aim of this study was to mimic a typical eating pattern, the biscuits provided were standardised based on weight, resulting in varying ACHOs which may cause differences in PPGR. Therefore, it would be valuable to run future studies matching the ACHO content in the biscuits. Moreover, although a postprandial duration of 4 h is sufficient to observe differences in our primary outcomes such as PPGR and PPIR, a longer duration may be necessary to detect variations in other parameters such as postprandial breath and lipid panel response. Lastly, future studies which may explore the underlying mechanism responsible for the improvement of PPGR following the consumption of BSG-containing biscuits, as well as the effect over long-term consumption, are required.

## 5. Conclusions

In conclusion, BSG-containing biscuits have a higher nutritive value compared to commercial wheat-based biscuits, which can help to regulate postprandial glycaemic response in individuals with MetS. This study posits BSG as a promising source of value-added ingredient, and fermentation as a potential method for biovalorising food by-products to enhance their health benefits.

## Figures and Tables

**Table 1:** Nutrient composition of test biscuits.

Nutrient (per 90 g Biscuits)	Control	ABSG	FBSG
Energy (kcal)	455 ± 5	440 ± 5	428 ± 5
Fat (g)	18.9 ± 0.0	20.0 ± 0.2	19.6 ± 0.2
Protein (g)	9.0 ± 0.1	10.4 ± 0.0	10.7 ± 0.0
ACHO (g)	61.2 ± 1.2	49.2 ± 0.9	47.1 ± 1.4
TDF (g)	1.9 ± 0.4	10.7 ± 0.4	10.4 ± 0.1
IDF (g)	1.2 ± 0.1	9.9 ± 0.3	9.3 ± 0.1
SDF (g)	0.7 ± 0.2	0.8 ± 0.2	1.1 ± 0.1
Phytic acid (g)	0.7 ± 0.0	0.9 ± 0.0	0.9 ± 0.0
Ash (g)	0.4 ± 0.2	1.0 ± 0.1	1.0 ± 0.2

Values are presented as mean ± SD. Control: control biscuit; ABSG: autoclaved brewers’ spent grain-containing biscuit; FBSG: fermented brewers’ spent grain-containing biscuit; ACHO: available carbohydrates; TDF: total dietary fibre; IDF: insoluble dietary fibre; SDF: soluble dietary fibre.

**Table 2:** Participant baseline characteristics.

Characteristics	Total	Male	Female
Number of subjects (*n*)	15	10	5
Age (years)	63 ± 10	61 ± 10	66 ± 12
WC (cm)	94.5 ± 12.0	96.6 ± 13.7	90.4 ± 6.5
HDL (mmol/L)	1.3 ± 0.1	1.1 ± 0.1	1.8 ± 0.2
Fasting glucose (mmo/L)	6.2 ± 0.4		
TG (mmol/L)	1.3 ± 0.2		
SBP (mmHg)	130.5 ± 18.5		
DBP (mmHg)	71.2 ± 11.2		
Insulin (mU/L)	10.0 ± 1.4		
HOMA-IR	2.61 ± 0.4		
T2DM medication (*n*)	5	3	2
Cholesterol-lowering medication (*n*)	9	6	3
Antihypertension medication (*n*)	6	3	3
**The number of MetS criteria met**			
3 (*n*)	8		
4 (*n*)	6		
5 (*n*)	1		

Values are presented as mean ± standard deviation or *n*. WC: waist circumference; HDL: high-density lipoprotein cholesterol; TG: triglyceride; SBP: systolic blood pressure; DBP: diastolic blood pressure; T2DM: type 2 diabetes mellitus; MetS: metabolic syndrome.

## Data Availability

The data presented in this study will be made available on request from the corresponding author. The data are not publicly available due to restrictions of informed consent and institutional guidelines.

## Supplementary Materials

The following supporting information can be downloaded at: https://www.mdpi.com/article/10.3390/nu16060909/s1. Figure S1: Change from baseline of (A) postprandial TC concentration. (B) Postprandial HDL-C concentration. (C) Postprandial LDL-C concentration. (D) Postprandial TG concentration; Figure S2: Change from baseline of (A) H_2_ concentration. (B) CH_4_ concentration; Figure S3: VAS score of subjective satiety assessment: (A) Hunger. (B) Desire to eat. (C) Prospective consumption. (D) Fullness; Table S1: Normalised AUC of TC, HDL-C and LDL-C, and positive iAUC and net iAUC of TG.
