# Supplementary material for: Impact of Brewers’ Spent Grain-Containing Biscuit on Postprandial Glycaemic Response in Individuals with Metabolic Syndrome: A Crossover Randomised Controlled Trial
Source: Nutrients. 2024 Mar 21;16(6):909. doi: 10.3390/nu16060909 (PMC10975460; doi:10.3390/nu16060909)
Supplement: Supplementary file 1 [file nutrients-16-00909-s001.zip › nutrients-2913391-supplementary.pdf]

Table S1. Normalized AUC of TC, HDL-C and LDL-C, and positive iAUC and net iAUC of TG.

Biomarker	Control	ABSG	FBSG
TC negative iAUC (mmol/L × min)	1657 ± 282[a]	1278 ± 192[a]	1394 ± 220[a]
HDL-C negative iAUC (mmol/L × min)	731 ± 108[a]	671 ± 78[a]	664 ± 65[a]
LDL-C negative iAUC (mmol/L × min)	2659 ± 440[a]	2445 ± 371[a]	2463 ± 294[a]
TG positive iAUC (mmol/L × min)	106 ± 16[a]	114 ± 23[a]	105.± 15[a]

Values are presented as mean ± standard error. *P* value was determined by repeated measured one-way ANOVA with post-hoc Bonferroni test. Different alphabet superscripts indicate a significant difference across the comparisons within the same row. Superscript a indicates the highest group across the row. Control: control biscuit; ABSG: autoclaved brewers' spent grain -containing biscuit; FBSG: fermented brewers' spent grain -containing biscuit. TC: total cholesterol; HDL-C: high-density lipoprotein cholesterol; LDL-C: low-density lipoprotein cholesterol; TG: triglyceride; AUC: area under the curve; iAUC: incremental area under the curve.

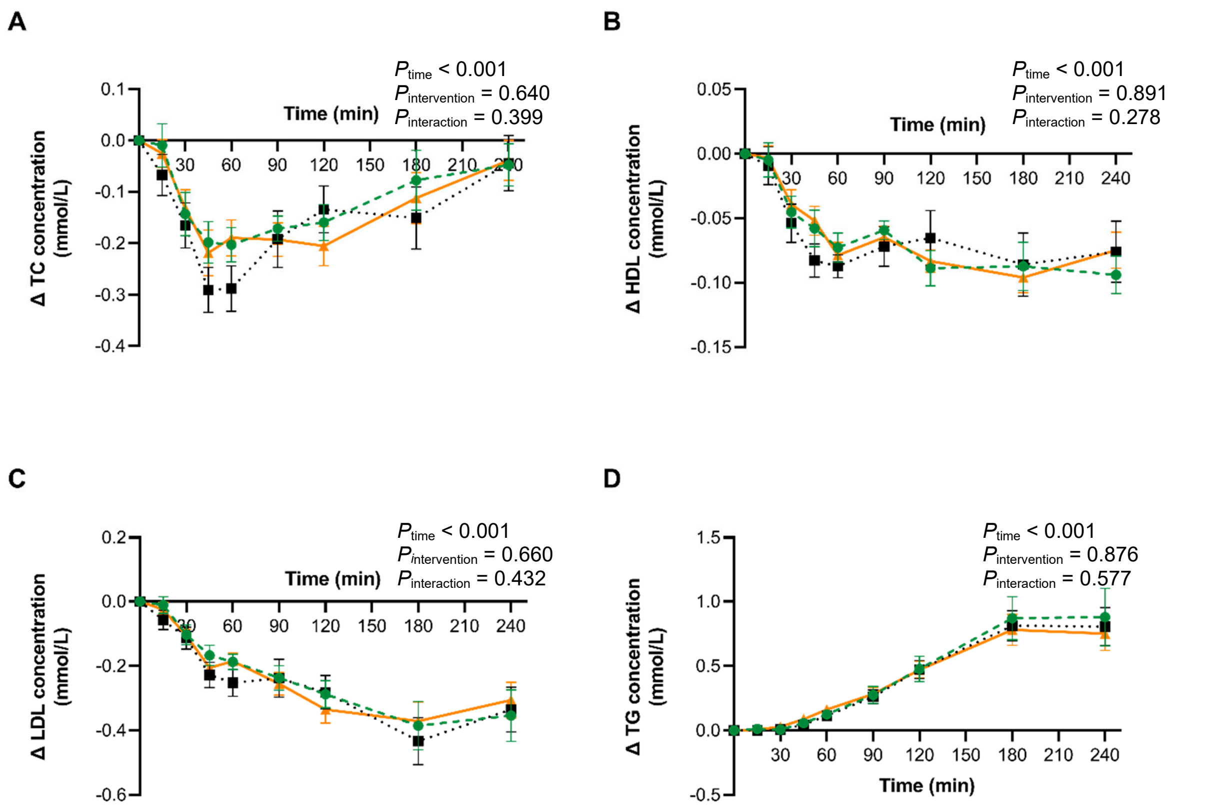

Figure S1. Change from baseline of **(A)** postprandial TC concentration. **(B)** postprandial HDL-C concentration. **(C)** postprandial LDL-C concentration. **(D)** postprandial TG concentration. Control is in black dotted lines, ABSG is in green dashed lines, FBSG is in orange solid lines. *P* value was determined by two-way repeated measures ANOVA with Wilcoxon test considering Bonferroni correction. Control: control biscuit; ABSG: autoclaved brewers' spent grain -containing biscuit; FBSG: fermented brewers' spent grain -containing biscuit. TC: total cholesterol; HDL: high-density lipoprotein cholesterol; LDL: low-density lipoprotein cholesterol; TG: triglyceride.

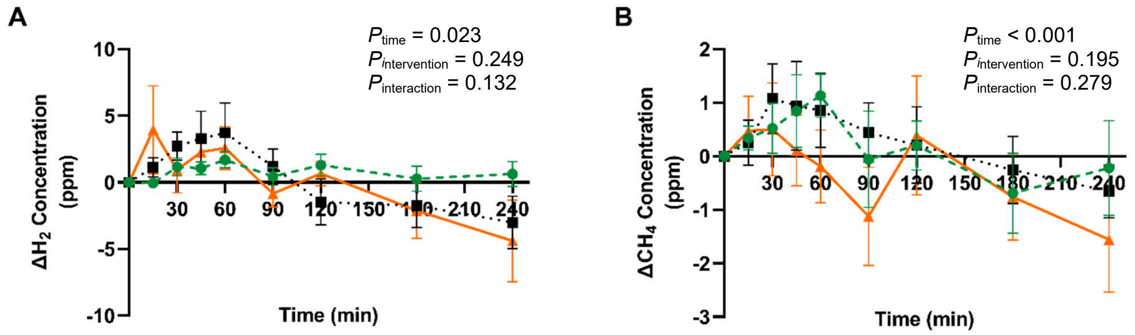

Figure S2. Change from baseline of **(A)** H_2 concentration. **(B)** CH_4 concentration. Control is in black dotted lines, ABSG is in green dashed lines, FBSG is in orange solid lines. *P* value was determined by two-way repeated measures ANOVA with Wilcoxon test considering Bonferroni correction. Control: control biscuit; ABSG: autoclaved brewers' spent grain -containing biscuit; FBSG: fermented brewers' spent grain -containing biscuit.

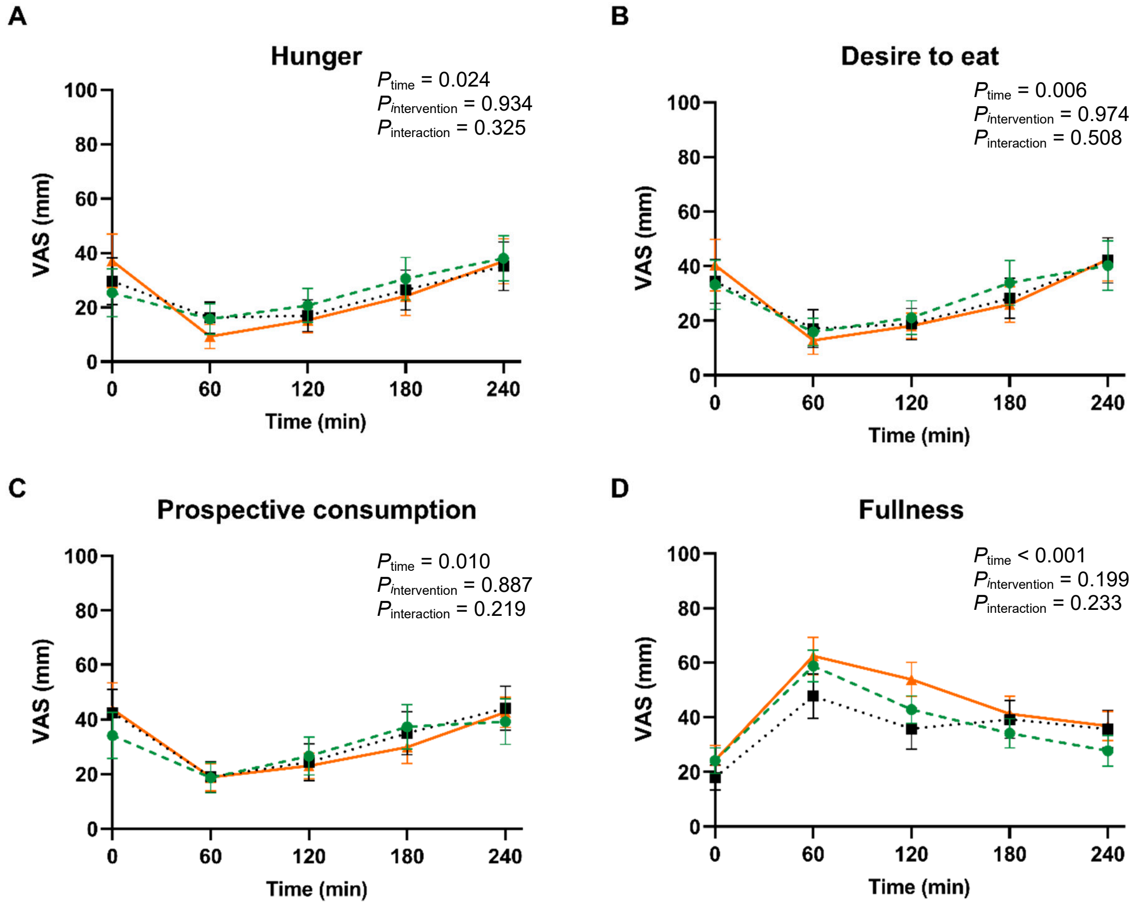

Figure S3. VAS score of subjective satiety assessment: **(A)** Hunger. **(B)** Desire to eat. **(C)** Prospective consumption. **(D)** Fullness. Control is in black dotted lines, ABSG is in green dashed lines, FBSG is in orange solid lines. *P* value was determined by two-way repeated measures ANOVA with Wilcoxon test considering Bonferroni correction. VAS: visual analogue scale; Control: control biscuit; ABSG: autoclaved brewers' spent grain -containing biscuit; FBSG: fermented brewers' spent grain -containing biscuit.
